# Preclinical evaluation of strasseriolides **A**–**D**, potent antiplasmodial macrolides isolated from *Strasseria geniculata* CF-247,251

**DOI:** 10.1186/s12936-021-03993-8

**Published:** 2021-12-05

**Authors:** Frederick Annang, Guiomar Pérez-Moreno, Caridad Díaz, Victor González-Menéndez, Nuria de Pedro Montejo, José Pérez del Palacio, Paula Sánchez, Scott Tanghe, Ana Rodriguez, Ignacio Pérez-Victoria, Juan Cantizani, Luis M. Ruiz-Pérez, Olga Genilloud, Fernando Reyes, Francisca Vicente, Dolores González-Pacanowska

**Affiliations:** 1 Fundación MEDINA, Avda. del Conocimiento 34, 18016 Armilla, Granada Spain; 2 Instituto de Parasitología y Biomedicina “López-Neyra”, Consejo Superior de Investigaciones Científicas (CSIC), Avda. del Conocimiento 17, Granada 18016 Armilla, Spain; 3 Department of Microbiology, New York University School of Medicine, 10016 New York, NY USA

**Keywords:** Natural products, Macrolides, Malaria, Drug development, Preclinical evaluation, Metabolic stability, Pharmacokinetics, In vivo efficacy, Drug–drug interaction, Cardiotoxicity

## Abstract

**Background:**

Malaria is a global health problem for which novel therapeutic compounds are needed. To this end, a recently published novel family of antiplasmodial macrolides, strasseriolides **A**–**D**, was herein subjected to in vivo efficacy studies and preclinical evaluation in order to identify the most promising candidate(s) for further development.

**Methods:**

Preclinical evaluation of strasseriolides **A**–**D** was performed by MTT-based cytotoxicity assay in THLE-2 (CRL-2706) liver cells, cardiotoxicity screening using the FluxOR™ potassium assay in hERG expressed HEK cells, LC–MS-based analysis of drug-drug interaction involving CYP3A4, CYP2D6 and CYP2C9 isoforms inhibition and metabolic stability assays in human liver microsomes. Mice in vivo toxicity studies were also accomplished by i.v. administration of the compounds (vehicle: 0.5% HPMC, 0.5% Tween 80, 0.5% Benzyl alcohol) in mice at 25 mg/kg dosage. Plasma were prepared from mice blood samples obtained at different time points (over a 24-h period), and analysed by LC-MS to quantify compounds. The most promising compounds, strasseriolides **C** and **D**, were subjected to a preliminary in vivo efficacy study in which transgenic GFP-luciferase expressing *Plasmodium berghei* strain ANKA-infected Swiss Webster female mice (n = 4–5) were treated 48 h post-infection with an i.p. dosage of strasseriolide **C** at 50 mg/kg and strasseriolide **D** at 22 mg/kg for four days after which luciferase activity was quantified on day 5 in an IVIS^®^ Lumina II imager.

**Results:**

Strasseriolides **A**–**D** showed no cytotoxicity, no carditoxicity and no drug-drug interaction problems in vitro with varying intrinsic clearance (CLint). Only strasseriolide **B** was highly toxic to mice in vivo (even at 1 mg/kg i.v. dosage) and, therefore, discontinued in further in vivo studies. Strasseriolide **D** showed statistically significant activity in vivo giving rise to lower parasitaemia levels (70% lower) compared to the controls treated with vehicle.

**Conclusions:**

Animal efficacy and preclinical evaluation of the recently discovered potent antiplasmodial macrolides, strasseriolides **A**–**D**, led to the identification of strasseriolide **D** as the most promising compound for further development. Future studies dealing on structure optimization, formulation and establishment of optimal in vivo dosage explorations of this novel compound class could enhance their clinical potency and allow for progress to later stages of the developmental pipeline.

**Supplementary Information:**

The online version contains supplementary material available at 10.1186/s12936-021-03993-8.

## Background

**Fig. 1:**
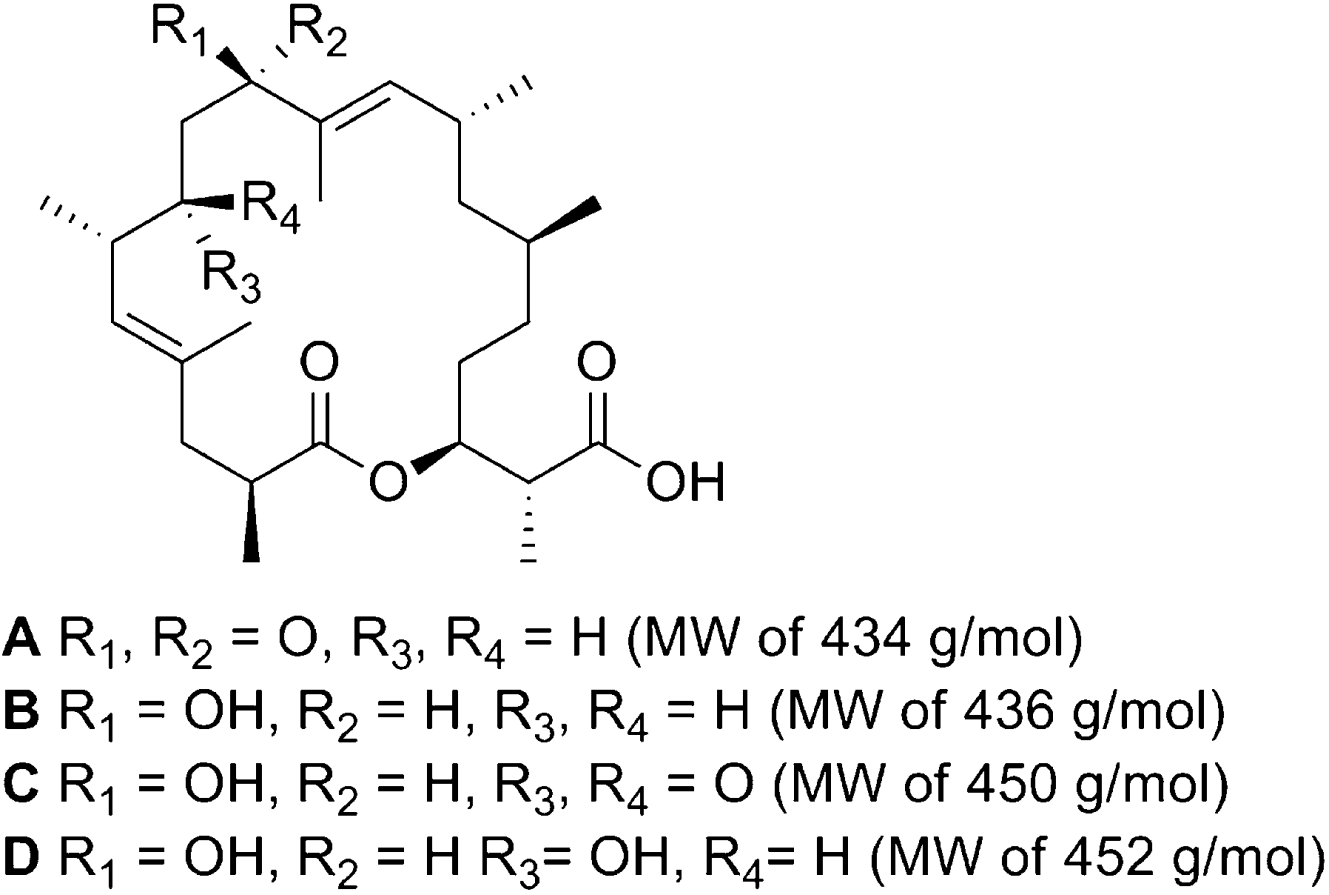
Structures of strasseriolides **A**–**D**

Malaria remains a global health problem causing mortality and morbidity in vulnerable populations such as in poor African countries. A recently described novel family of four macrolides, strasseriolides **A**–**D** (Fig. 1), isolated from the fungus *Strasseria geniculata* CF-247,251, exhibits IC_50_ values between 0.013 and 9.810 µM against *Plasmodium falciparum* strain 3D7 whole parasites and is active against chloroquine resistant *P. falciparum* strain Dd2 parasites [1]. The discovery of novel active compound(s) marks a key milestone in the early stages of the complex, capital-intensive drug discovery process [2]. Most of the cost of a drug discovery programme is usually inherent to the extremely high attrition rate of molecules that never see the daylight in the developmental process. In order to minimize this cost, it is critical to integrate early in vitro and in vivo preclinical experiments at the discovery stage [3, 4]. Such experiments will provide relevant preliminary data that can aid in predicting with reasonable accuracy the potential of newly discovered compounds to reach later stages of the developmental pipeline [3, 4]. This is especially important in the case of natural products since compounds from this source may have an unusually complex chemistry with peculiar and hidden pharmacokinetic challenges. Bearing these considerations in mind, the recently discovered antiplasmodial natural macrolides (strasseriolides **A**–**D**) [1] were subjected to a series of preclinical evaluation experiments that included, in vitro cytotoxicity in immortalized THLE-2 liver cells, cardiotoxicity screening using the FluxOR™ potassium assay in hERG expressed HEK cells, drug-drug interaction and metabolic stability in liver microsomes, and in vivo toxicity, PK and efficacy studies in mice. The data obtained from these studies identified strasseriolide **D** as the most promising compound that merits further investigation.

## Methods

### Ethics statement

The maintenance and care of mice used in this work were conformed to institutional guidelines (CETS n° 123). Protocols to perform studies on in vivo toxicity in mice and quantification of compounds in mice plasma were approved by the Instituto de Parasitología y Biomedicina “López Neyra” (CSIC) Ethical Committee. In vivo efficacy was monitored at New York University School of Medicine (US). This study was carried out in accordance with the recommendations in the Guide for the Care and Use of Laboratory Animals of the National Institutes of Health (US). The protocol was approved by the Institutional Animal Care and Use Committee of New York University School of Medicine, which is fully accredited by the Association for Assessment and Accreditation of Laboratory Animal Care International [5].

### Reagents and chemicals

Reduced nicotinamide adenine dinucleotide phosphate tetrasodium salt (NADPHNa_4_), testosterone, diclofenac and dextromethorphan (standard Cytochrome P450 probe substrates), cortisone and levallorphan (LC–MS–MS internal standards), ketoconazole, quinidine and sulfaphenazole (Cytochrome P450 control inhibitors) were obtained from Sigma Aldrich (St. Louis, MO, USA). 4’-hydroxydiclofenac ^13^C_6_ (LC–MS–MS internal standard) was purchased from Toronto Research Chemical (Toronto). Human liver microsomes (HLM) mixed gender pool of 22 were purchased from BD Gentest Corp (Bedford, MA). Materials related to the chemistry part of the experiments include acetonitrile (gradient grade, Merck KGaA), formic acid (reagent plus, Sigma Aldrich) and milli-Q water 18.2 Mega Ohm (Milli-Q gradient system, Merck Millipore).

### In vitro cytotoxicity assay

Cytotoxicity of strasseriolides **A**–**D** was determined against immortalized THLE-2 (CRL-2706) liver cells by the MTT assay. The cells were first seeded in 96-well plates at 1 × 10^4^ cells/well in 200 µL MEM culture medium and incubated at 37 °C in 5% CO_2_ for 24 h. The spent medium was replaced with fresh 200 µL medium containing 1 µL of compounds and controls. The controls were, 8 mM methyl methanesulfonate (MMS, positive control), DMSO 0.5% (negative control) and the standard drug doxorubicin. Compounds were tested in triplicate using twelve-point ½ dose-response curves with maximum concentrations at 50 µM. The cells were further incubated with the compounds for 72 h. After incubation, the MTT solution was prepared at 5 mg/mL in PBS and then diluted to 0.5 mg/mL in medium without phenol red. The spent medium was then replaced with 100 µL of MTT solution and the plates gently shaken and incubated at 37 °C in 5% CO_2_ for 3 h. The supernatant was then carefully removed without disturbing the cells and 100 µL DMSO added. The plates were gently shaken to solubilize the formed formazan and absorbance measured at 570 nm in a Victor2^TM^ multireader (Perkin Elmer, USA). The percentage inhibition of cell growth was computed by the formula:

$$Percentage \, inhibition=\left[1-\left(\frac{{Abs}_{well}-{Abs}_{neg}}{{Abs}_{pos}-{Abs}_{neg}}\right)\right] \times 100$$

where *Abs*_well_ is the absorbance measured per specific well, and *Abs*_pos_ and *Abs*_neg_ are the average absorbance measured for the positive and negative controls respectively.

### In vitro cardiotoxicity screening

Cardiotoxicity screening of strasseriolides **A**–**D** was performed in hERG expressed HEK cells using the FluxOR™ potassium assay. The FluxOR™ potassium assay was performed on a FLIPR TETRA (Molecular Devices) as outlined in the product information sheet from Invitrogen. As directed by the kit, the Powerload™ concentrate and water-soluble probenecid were respectively added in the first step to enhance the dye solubility and retention, after which the FluxOR™ dye was added and mixed. The FluxOR™ loading buffer (165 mM NaCl, 4.5 mM KCl, 2 mM CaCl_2_, 1 mM MgCl, 10 mM HEPES, 10 mM Glucose) was adjusted to a pH of 7.4. Spent medium from cells previously seeded as described above for the MTT assay, was removed and replaced with 80 µL loading buffer containing the FluxOR™ dye mix. The dye was removed after 60 min incubation at room temperature and the plates subsequently washed once with assay buffer. The compounds were dissolved in DMSO after which 2 µL of compound solution was added to 398 µL assay buffer using a Biomek liquid handling unit (Beckman Coulter). Hundred microlitres (100 µL) of the diluted compounds were added to the cells and the plates incubated for 30 min at room temperature (23–25 °C) to allow equilibration of the compounds. The thallium stimulation buffer (Tl_2_SO_4_ + K_2_SO_4_) was prepared according to the manufacturer´s instruction and injected into the plates on the FLIPR TETRA, to allow kinetic analysis from time zero (t_0_) to time 120 s (t_120_). In each well, the average of values for 2-5 s was used as background and that for 90-92 s was used as maximum channel activity. The ratio of maximum channel activity to background values recorded in each well was used for calculating the percentage of inhibition of each compound with respect to the control wells in which no compounds were added. The compounds were tested in triplicate using twelve-point ½ dose-response curves with maximum concentrations at 50 µM.

### Cytochrome P450 inhibition assay (drug–drug interaction)

The Cytochrome P450 inhibition assay was performed using substrates for the CYP3A4, CYP2D6 and CYP2C9 isoforms of the enzyme [6, 7]. The 96-well ABgen-0765 plates used in the assay were first pretreated by being washed with acetonitrile, sonicated for 5 min and rinsed with deionized water, after which they were centrifuged upside down to dryness and kept warmed at 37°C in a water bath before used. As the test compounds were initially dissolved in DMSO, the final DMSO content of each reaction mix was established at 0.35% to minimize its interference with the reactions. Two microlitres (2 µL) of compounds were pre-incubated with 98 µL of cofactor/buffer solution at 37°C for 10 min (resulting final conc. of NADPH/phosphate buffer was 1 mM/100 mM at pH 7.4 when HLM/substrate was added). The reaction was then initiated by the addition of 100 µL of HLM/substrate solution (at final protein concentration of 0.25 mg/mL). For CYP3A4, CYP2D6 and CYP2C9 enzyme isoforms, 50 µM testosterone, 22 µM dextromethorphan and 10 µM diclofenac were respectively used as substrates in the HLM/substrate solution. No inhibitor and inhibitor controls (i.e. ketoconazole for CYP3A4, quinidine for CYP2D6 and sulfaphenazole for CYP2C9) were included in all plates at the compound addition stage. The reactions were incubated for 15, 30 and 45 min with shaking at 180 Hz, for CYP3A4, CYP2D6 and CYP2C9 respectively, after which they were quenched by adding 90 µL acetonitrile containing internal LC–MS–MS standards (0.166 µM cortisone, 0.314 µM 4’-hydroxydiclofenac ^13^C_6_ and 0.212 µM levallorphan for CYP3A4, CYP2D6 and CYP2C9, respectively). The plates were gently mixed on a microtiter shaker for 1 min and centrifuged at 4000 rpm for 20 min at 4 °C. Fifty microlitre (50 µL) supernatants were then transferred from the reaction wells to new 96-well round bottom plates and 25 µL of 0.3% formic acid/water added. The new plates were centrifuged at 4000 rpm for 15 min at 4 °C, sealed and placed in a mass spectrometer with an auto-sampler. LC–MS–MS analysis was performed to quantify the relative amounts of 6β-hydroxy-testosterone (CYP3A4), dextrorphan (CYP2D6) or 4’-hydroxy-diclofenac (CYP2C9) products formed from the respective substrates. A triple quadrupole mass spectrometer (AB SCIEX API4000) coupled to an Agilent 1290 HPLC system (Agilent Technologies, USA) was used for the quantitative analysis. Analysis was performed on a Discovery HS C18 (50 mm, 2 mm, 3 μm) column (Supelco, Torrance, CA) at 25 °C with mobile phase solvents A (water/acetonitrile 90/10 (v/v) + 0.1% formic acid), B (90/10 acetonitrile/water + 0.1% formic acid) and flow rate of 1 mL/min. Mobile phase B was initially held for 0.5 min at 10%, then increased linearly to 45% during a period of 1.50 min, after which it was maintained at this percentage for 0.30 min, and increased linearly again to 95% during a period of 0.30 min. It was then held at 95% for 0.50 min, before bringing it down to 10% B in 0.10 min and maintained there for 0.61 min to re-equilibrate the column. In all, the total run time was 3.51 min. The mass spectrometer was operated with an atmospheric pressure chemical ionization (APCI) probe in positive mode using multiple reaction monitoring (MRM) scanning mode. Integration of reaction product and internal standard peak areas was performed using the Analyst® software version 1.5.2. Each compound was tested in triplicate using ten-point ½ dose-response curves with maximum concentrations at 83 µM.

### Drug metabolic stability assay in human liver microsomes

Pretreatment of the 96-well ABgen-0765 reaction plates was the same as previously described above. Two microlitres (2 µL) of compounds (previously dissolved in DMSO) were pre-incubated with 98 µL of cofactor/buffer solution (0.1 M phosphate buffer of pH 7.4, 100 mM K_3_PO_4_, 3.3 mM MgCl_2_, 3.3 mM G6P, 1.3 mM NADPH, and 0.4 u/mL G6PDH) at 37 °C for 10 min. The reactions were then initiated by the addition of 100 µL of HLM solution (at final protein concentration of 1 mg/mL), after which they were stopped at pre-determined incubation times of 0, 10, 15, 30, 45 and 60 min by adding 90 µL acetonitrile containing internal LC–MS–MS standards and shaking at 180 Hz. The samples were centrifuged to sediment the protein precipitates and the supernatants were analysed by LC–MS–MS as previously described above. The integration of each analyte (the ionized metabolite of each compound) and internal standard peak area was performed by the Analyst® software version 1.5.2 and used to calculate the percentage of remaining compound at each incubation time. The natural logarithm of the percentage of the remaining parent compound was plotted against the incubation time and used to calculate the half-life by the following equation:

Half-life (T ½ min) = 0.693/k, where k is the slope of a plot of the natural log of the percentage parent compound remaining vs. time.

The intrinsic clearance (CLint) was determined by the equation: CL_int_

$$=\frac{Ln 2}{{\text{t}}_{\frac{1}{2}\left(\text{m}\text{i}\text{n}\right)}}* \frac{volume \, incubation \left(mL\right)}{microsomal \, protein \left(mg\right)}*45\left(MPPGL\right)\frac{1500 \, g \, human \, liver}{70 \, kg \, human \, body \, weight}j$$

Units for CLint were expressed as mL/min/mg microsomal protein and MPPGL = mg microsomal protein per gram liver.

### In vivo toxicity and quantification of compounds in mice plasma by LC–MS–MS

Three Balb/C mice (6–8 weeks old) were intravenously injected with 25 mg/kg of strasseriolide **D** dissolved in 200 µL of vehicle (0.5%HPMC, 0.5% Tween 80 and 0.5% Benzyl alcohol in water). Blood samples were collected by pricking the jugular vein on the leg of each mouse at time points 1.5 (T 1.5 h), 5 (T 5 h) and 24 h (T 24 h). From the harvested blood, 50 µL plasma were prepared for all the samples and stored at -80 °C until needed. A previously developed LC–MS–MS method (see supporting information) was used to determine the plasma concentration of strasseriolide **D** as described below.

Ten millimolar (10 mM) DMSO stocks of strasseriolide **D** (the test compound) and strasseriolide **A** (used as internal standard,) were prepared. From the strasseriolide **D** stock, a 59.7 µM (27,000 ppb) working concentration was made in DMSO and an aliquot of this was serially diluted (½) in reconstitution solution (1:1 of acetonitrile: H_2_O with 0.1% formic acid) to create a six-point calibration curve in triplicate. Three different quality control (QC) concentrations of strasseriolide **D** at 59.7, 0.74, and 0.25 µM (i.e. 27,000 ppb, 333 ppb and 111 ppb) were also prepared in duplicate. From the strasseriolide **A** (internal standard) stock, a 6.2 µM (2700 ppb) working concentration was prepared in DMSO.

Blank mouse plasma (prepared from non-injected mouse) was retrieved from the freezer (− 80 °C) and kept on ice to thaw. The sample was vortexed adequately and 15 µL aliquots transferred into 0.7 mL Eppendorf tubes after which 35 µL of Milli-Q water were added and vortexed. Triplicate sets of the diluted plasma were then respectively spiked with 2 uL of each concentration of the calibration curves of strasseriolide **D**. Another triplicate set of the diluted plasma was spiked with 2 µL of each concentration of the standard QC solutions. All the above samples were also spiked with 2 µL of strasseriolide **A** (the internal standard)). To serve as blank samples, a fresh triplicate set of diluted plasma were spiked with 4 µL of DMSO. Non-matrix samples were also prepared by spiking 100 µL aliquots of reconstitution solution with 2 µL of the standard QC solutions and 2 µL of internal standard.

Fifteen microlitres (15 µL) plasma from the various time points of the strasseriolide **D-**injected mice, were respectively transferred into 0.7 mL Eppendorf tubes. To each sample, 37 µL of Milli-Q water were added, vortexed and spiked with 2 µL of strasseriolide **A** (internal standard). Two hundred microlitres (200 µL) of ice-cold acetonitrile were added to all of the matrix (plasma-containing) samples and centrifuged at 13,300 rpm for 15 min at 4 °C. Subsequently, 180 µL aliquots were carefully transferred (without disturbing the protein precipitate) from each tube into clean HPLC vials and evaporated to dryness in an EZ2 Genevac at room temperature. The dried samples were then reconstituted in 40 µL of reconstitution solution by vortexing. The samples were placed in an auto-sampler drawer for LC–MS–MS analysis and quantification of strasseriolide **D** (the test compound) in mice plasma.

**Fig. 2:**
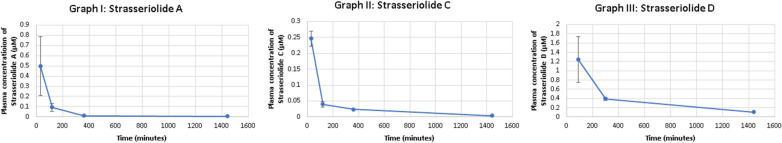
Compound concentration vs. time graphs of strasseriolides **A, C **and** D** used in extrapolating their pharmacokinetic (PK) parameters. For each compound, triplicate mice were injected with I.V. dosage of 25 mg/Kg compound and LC–MS–MS used to quantify compound concentrations in plasma collected at 3-4 different time-points within a 24-hour period. Graphs I, II and III show concentration vs. time relationship for strasseriolides **A**, **C** and **D** respectively

Similar sample preparation and LC–MS–MS analysis were respectively performed for mice intravenously injected with strasseriolides **A** and **C** as test compounds and mice blood samples collected at 30 min (T 30 M), 2 h (T 2 h), 6 h (T 6 h) and 24 h (T 24 h). In the case of strasseriolide **B**, the three mice injected with this compound died about eight minutes after their injection, hence, blood samples were immediately collected, and plasma prepared for analysis as previously described. For strasseriolides **A**, **B** and **C** as test compounds; strasseriolides **C**, **D** and **B** were respectively used as internal standards. After LC–MS–MS analysis, the plasma concentration of compound vs. time data was determined for each strasseriolide. This data was fed into the *PK solver 2.0* add-in program in an Excel file with “non-compartmental analysis of plasma data after intravenous bolus input” [8], to compute the PK parameters of each tested compound and to plot plasma concentration vs. time curves from which the PK (pharmacokinetic) parameters were computed for the strasseriolides (Table 2; Fig. 2).

Animals were observed individually at 15, 30 and 60 min after dosing and periodically during the first 24 h for any clinical signs of toxicity or mortality. The toxicity evaluation was carried out by observing behaviour parameters, including the Grimace scale parameters. The mice were sacrificed, and the main organs were examined by visual inspection during autopsy.

### In vivo efficacy evaluation of strasseriolides C and D in mice

In vivo efficacy experiments were performed at the Anti-infectives Screening Core Services facility of the New York University School of Medicine [5]. The transgenic *P. berghei* line 676m1cl1 parasite (PbGFP-Luccon) expressing the fusion GFP (mutant 3) and firefly luciferase (LucIAV) proteins generated in the reference clone of ANKA strain cl15cy1 was used [9]. Swiss Webster female mice were infected with transgenic *P. berghei* line 676m1cl1 (PbGFP-Luccon) ANKA-infected 10³ red blood cells. Treatment was then started 48 h post-parasite infection by i.p. dosage of 22 mg/kg of strasseriolide **D** and 50 mg/kg of strasseriolide **C**. Vehicle only (PBS 2% methylcellulose, 0.5% Tween 80) and 20 mg/kg chloroquine were used as negative and positive controls. The infected mice were treated for four days, after which luciferase activity of the parasites was quantified 5 to 10 min after i.p. injection of 150 mg/kg D-Luciferin Potassium-salt (Goldbio) dissolved in PBS using an IVIS® Lumina II imager on the fifth day.

### Data analysis and statistics

**Fig. 3:**
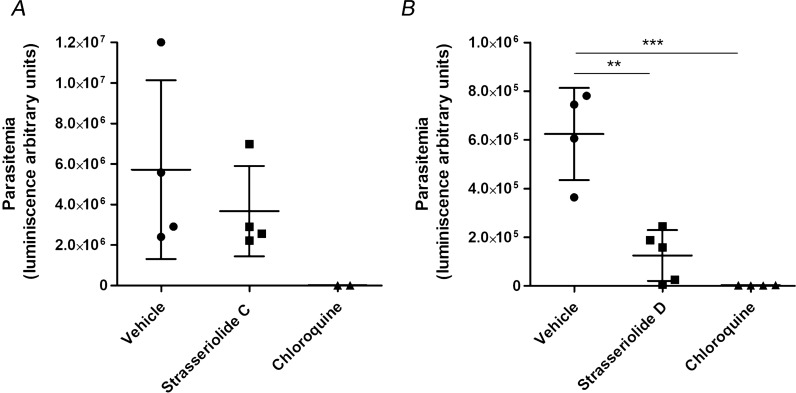
Representation of in vivo mice efficacy after 4 day strasseriolides **C** and **D** treatments. Mice were infected with the transgenic *P. berghei* line 676m1cl1 and treated at 48 h post-infection with 50 mg/kg of strasseriolide **C** (four replicates, graph A) and 22 mg/kg of strasseriolide **D** (five replicates, graph B) for 4 days. The vehicle and 20 mg/kg chloroquine were used as controls. The asterisks show significant differences calculated by the Student’s t test. **p < 0.01, ***p < 0.001, vs. the vehicle

The *Genedata Screener* online application (Genedata AG, Basel, Switzerland) was used to compute IC_50_ values. The Smart Fit strategy with Hill model was selected as the choice for curve fitting. Where applicable, data are presented as mean ± standard deviation (SD). In Fig. 3, the significant differences between the vehicle controls and test compounds were calculated by the Student’s t test.

## Results

In preclinical safety profiling of strasseriolides **A**–**D**, the in vitro toxicity assay in immortalized THLE-2 (CRL-2706) liver cells showed no toxicity at the maximum concentration tested (IC_50_ > 50 µM) for all four compounds. Cardiotoxicity screening in hERG expressed HEK cells using the FluxOR™ potassium assay also indicated that all four compounds had no effect on the inward rectifying potassium ion channel at the maximum concentrations tested (IC_50_ > 50 µM). Cytochrome P450 inhibition experiments performed using CYP3A4, CYP2D6 and CYP2C9 isoforms by incubating human liver microsomes (HLM) with the respective substrates of these enzyme isoforms demonstrated that at concentrations ≤ 43 µM, none of the compounds inhibit the cytochrome isoforms investigated (i.e. IC_50_ > 43 µM). The respective metabolic stabilities obtained also showed strasseriolides **A** and **B** to have high liver microsome clearance, while **C** and **D** had medium and low clearance, respectively (Table 1).

**Table 1:** Metabolic stability of strasseriolides **A–D**

	T_1/2_	Cl_int_	Clearance
Strasseriolide	(min)	(mL/min/mg)	Category
A	6.47 ± 1.05	103.28 ± 22.6	High
B	8.7 ± 2.4	76.81 ± 9.8	High
C	15.91 ± 1.15	42.00 ± 1.92	Medium
D	>60	>11.13	Low

Further, a preliminary pharmacokinetic (PK) study of strasseriolide **A**–**D** was performed in mice using an in-house developed LC–MS method (see Additional file 1). The data from this experiment was used to construct plasma concentration vs. time graphs for the compounds (Fig. 2), and was also fed into a *PK solver 2.0* add-in program in excel and a “non-compartmental analysis of plasma” was performed to extrapolate the respective pharmacokinetic (PK) parameters, a summary of which are shown in Table 1 (for details of PK parameters, see Additional file 1: Tables S3a, S5a, S6a) [8].

The three mice in each group respectively injected with strasseriolides **A**, **C** and **D**, survived until 24 h post-i.v. injection when they were euthanized for analysis. At 15, 30 and 60 min and periodically during the first 24 h of injection, toxicity evaluation of these compounds was performed by observing different parameters of their behaviour. The compounds did not give rise to any signs of toxicity or mortality in mice. Furthermore, the main organs were inspected and did not show apparent damage. However, the three mice injected with strasseriolide **B**, started showing aggravated signs of toxicity (significantly reduced mobility) almost immediately after injection. The conditions of these mice deteriorated very rapidly until they all died just under eight minutes post-i.v. injection. Blood samples were immediately collected from the dead mice and processed to obtain the plasma for analysis. The only recorded plasma concentration of strasseriolide **B** was 19.6 ± 1.2 µM (Table 2). Strasseriolide **B** was further tested at lower dosages of 10, 5 and 1 mg/Kg, but all resulted in death of the mice injected. This confirmed that strasseriolide **B** was extremely toxic to mice and, therefore, its usage in further in vivo experiments was discontinued.

**Table 2:** Summary PK data of strasseriolides **A–D**

Strasseriolides	T_initial_ (h)	Estimated PK parameter					
		Plasma Conc. (µM)		T_1/2_ (h)	AUC_0−t_ (µM x h)	MRT (h)	Vz_obs (L/kg)
		@T_initial_	@T_24 h_				
A	0.5	0.498 ± 0.29	<0.0048	4.4	1.14	2.98	312.4
B*	0.13	19.6 ± 1.2	–	–	–	–	–
C	0.5	0.246 ± 0.023	0.0035 ± 0.0009	6.3	0.766	4.7	636.8
D	1.5	1.247 ± 0.023	0.107 ± 0.005	7.3	10.06	8.0	51.9

Where T_1/2_ is the apparent terminal elimination half-life time, AUC_0-t_ is the area under the concentration vs. time curve (calculated as sum of AUCs using linear trapezoidal summation from time 0 to the last measurable data point), MRT is the mean retention time, and Vz_obs is the plasma distribution volume

*Only T_initial_ and the Plasma Conc @ T_initial_ were recorded as all the mice injected with this compound died under 8 min

As shown in Table 2, the non-toxic strasseriolides **A**, **C** and **D** were detectable in mouse plasma at 24 h post-injection with mean concentrations of < 0.0048, 0.0035 ± 0.0009 and 0.107± 0.005 µM, respectively. The area under the curve (AUC_0-t_) for strasseriolide **D** (10.06 µM x h) was thirteen times that for **C** (0.766 µM × h) and nine times that for **A** (1.14 µM × h). Further, the mean retention times (MRT) and half-life (T_1/2_) for strasseriolide **D** (8 and 7.3 h respectively) were significantly higher than that obtained for **C** (4.7 and 6.3 h respectively) and **A** (2.98 and 4.4 h, respectively). The theoretical Cmax achievable with the 25 mg/Kg drug dosage (i.e. 0.5 mg drug in 200 µL vehicle) was calculated at 550–570 µM for all the four compounds (i.e. 250,000 ng/mL, see supporting information). This value was obtained with the assumption that total plasma volume of mouse was 1.8 mL [10] and that the four compounds were completely soluble in both the vehicle and in mouse plasma.

Considering the absence of in vivo toxicity of strasseriolides **C** and **D** to mice and their potent in vitro activity against *Plasmodium falciparum* parasites, strain 3D7, with IC_50_ values of 0.123, and 0.128 µM, respectively [1], the two compounds were further investigated in preliminary in vivo efficacy experiments. Strasseriolide **D** significantly lowered parasitaemia in vivo, as indicated by the measurement of Arbitrary Luminescence Units in comparison to the control vehicle (1.25 × 10^5^ ± 1.04 × 10^5^ vs. 5.07 × 10^5^ ± 3.08 × 10^5^, p < 0.01) (Fig. 3B, Additional file 1: Table S8) while strasseriolide **C** exhibited moderate in vivo activity, although not statistically significant due to data dispersion (3.66 × 10^6^ ± 2.23 × 10^6^ vs. 5.72 × 10^6^ ± 4.43 × 10^6^) (Fig. 3A, Additional file 1: Table S7).

## Discussion

A previously reported potent family of four novel antiplasmodial macrolides, strasseriolides **A**–**D** with respective IC_50_ values of 9.810, 0.013, 0.123, and 0.128 µM, was obtained by bioassay-guided isolation from an extract produced by the fungus *Strasseria geniculata* CF-247,251 [1]. In the current study, the compounds were subjected to preclinical evaluation in order to identify the most promising candidates for further development.

The establishment of potential drug–drug interactions data is a key requirement of drug development since abrupt alterations in drug metabolism (or transport) may lead to significant changes in safety and efficacy. The potential drug–drug interaction risks of the newly discovered compounds were assessed by a cytochrome P450 inhibition assay determined by LC–MS analysis, which showed all the four compounds to have IC_50_ values > 43 µM when incubated with CYP3A4, CYP2D6 and CYP2C9 isoforms [11], thus all compounds have low risk of potential drug–drug interactions. The four strasseriolides were also tested in hERG expressing HEK cells and were found to have no effect on this inward rectifying ion channel (IC_50_ values > 50 µM). Hence, no risks involving possible interference with the normal functioning of the heart were identified. This is a key part of the risk assessment criteria for new chemical entities since drug-induced ECG effect and associated long QT syndromes may lead to increased risk of “torsade de pointes” ventricular tachyarrhythmia and sudden death [12, 13]. The compounds were also non-cytotoxic against immortalized THLE-2 liver cells.

Additionally, both prolonged unnecessary systemic exposure and insufficient exposure to potential drugs may give a poor PK feature leading to their termination from a developmental portfolio. The metabolic stabilities of the four new macrolides (**A**–**D)** were thereby tested in human liver microsomes and ranked by intrinsic clearance (CLint), as high, high, medium and low respectively. This indicates that strasseriolides **A** and **B** are the most metabolically unstable, whilst the low clearance of strasseriolide **D** is indicative of a possible long systemic exposure which although being a positive feature for potential in vivo efficacy, may present possible liabilities in systemic toxicity due to the prolonged exposure. Strasseriolide **C**, which has medium clearance, may have enough metabolic stability for potential in vivo efficacy with lower possible liabilities of toxicity. Follow-up in vivo experiments in mice for preliminary toxicity assessment and PK profiling showed strasseriolides **A**, **C** and **D** to be very tolerable at the dosage of 25 mg/Kg (non-toxic) in vivo; no signs of toxicity were identified, and animals survived until 24 h post-intravenous injection, when they were euthanized for organ analysis. The non-toxic strasseriolides **A**, **C** and **D** could be further evaluated for their toxicity in mice over a longer period of time (e.g. 14 days) in follow up experiments. Strasseriolide **B**, the most potent antiplasmodial compound in vitro (IC_50_ of 0.013 µM againt *P. falciparum* 3D7 parasites), turned out be extremely toxic in vivo, killing almost immediately all mice upon i.v. injection at dosages of 25, 10, 5 and even 1 mg/kg. Although this compound showed no in vitro toxicity against THLE-2 and HepG2 liver cells as already described [1], its plasma concentration was 19.6 µM ± 1.2 µM after 8 min of i.v. injection at 25 mg/kg. The sudden death of mice injected with this compound suggests rapid systemic toxicity, although the exact cause of rapid death remains to be established.

The PK parameters of the three non-toxic strasseriolides (**A**, **C** and **D**) indicated very low plasma concentrations in the first 30 min (strasseriolides **A** and **C**) or 90 min (strasseriolide **D**) after administration taking into account the initial expected concentration of 553-570 µM (250,000 ng/mL). Moreover, high plasma distribution volumes, i.e. 312.4, 636.8 and 51.9 L/kg respectively, were obtained for all the three compounds, i.e. **A**, **C** and **D** (Table 1), suggesting a possible high tissue distribution. The low plasma concentrations of the three compounds could also be explained by their possible high renal clearance. There may also be solubility issues, since preliminary (unpublished) data shows that at 25 mg/Kg dosage (i.e. at concentration of about 5.53 mM in 200 µL vehicle), these compounds are only about 35% soluble in the formulation vehicle used in this study. The low plasma concentrations in the cases of strasseriolides **A** and **C** may also be explained by their high-medium metabolic clearance as previously indicated by the in vitro metabolic stability analysis (Table 2).

A careful consideration of the preclinical characterization data as a whole unequivocally presented strasseriolides **C** and **D** as the most promising compounds, hence their preliminary evaluation in mouse in vivo efficacy experiments. Promising results were obtained, especially for strasseriolide **D** which showed superior effect in vivo according to a significantly lower parasitaemia level (70% lower) as opposed to **C** (30% lower), when compared to the controls treated with vehicle only (Fig. 3). Since strasseriolides **C** and **D** showed moderate and significant efficacy respectively in vivo, both compounds could be considered for further investigation to improve their efficacy in secondary in vivo studies [14, 15]. The results obtained for strasseriolide **D** were the most promising, since at the lower dosage of 22 mg/kg, this compound showed a superior effect in vivo than the 50 mg/kg dosage of strasseriolide **C**. A closer look at the raw arbitrary luminescence units obtained for strasseriolide **D** in replicates 4 and 5 (2.56 × 10^4^ and 6.93 × 10^3^ respectively) makes this compound even more interesting as the data approaches to that obtained for chloroquine (3 × 10^3^) at a similar dosage of 20 mg/kg (Additional file 1: Table S8). Strasseriolide **D** warrants secondary in vivo evaluation in order to explore higher dosage regimes in both oral and i.p. drug administration routes to determine its ED_50_ and ED_90_ values. Studies aimed at exploring different formulation approaches could also lead to potential enhancement of the in vivo efficacy of the compound as it was 35% soluble in the formulation vehicle used in this study. Additionally, although it seems obvious that the hydroxyl group at R1 (Fig. 1) in strasseriolides **C** and **D** plays a key role in the in vitro potency [1], a future medicinal chemistry program may allow for the generation of novel analogues of this new family. Thereby, structure-activity (SAR) optimization studies may enhance their potency and identify the exact reason for the extreme toxicity of strasseriolide **B**.

## Conclusions

A novel family of potent antiplasmodial macrolides, strasseriolides **A**–**D**, recently isolated from extracts of *Strasseria geniculata* CF-24,725 (1), was subjected to preclinical evaluation, leading to the identification of strasseriolide **D** as the most promising compound with the best PK parameters and in vivo efficacy (without toxicity), for further development. Exploring different formulations and dosage regimes to further establish the therapeutic windows of strasseriolide **D** are also called for in order to improve in vivo efficacy.

## Supplementary information

**Additional file 1: Fig. S1.** Calibration curve of test strasseriolide **B** (i.e. peak area ration of strasseriolide **B**:**C** vs concentration of **B**). **Table S1.** Calibration curve table of strasseriolide **B**. **Table S2.** QC table used to calculate the precision and accuracy of the method. **Table S3a.** Strasseriolide **A** quantification in mouse plasma (i.v. dosage of 25 mg/kg). **Table S3b.** Non-compartmental analysis of strasseriolide **A** in mouse plasma (after i.v. bolus input). **Table S4.** Strasseriolide **B** quantification in mouse plasma (i.v. dosage of 25 mg/kg). **Table S5a.** Strasseriolide **C** quantification in mouse plasma (i.v. dosage of 25 mg/Kg).  **Table S5b.** Non-compartmental analysis of strasseriolide **C** in mouse plasma (after i.v.  bolus input). **Table S6a.** Strasseriolide **D** quantification in mouse plasma (i.v. dosage of 25 mg/Kg). **Table S6b.** Non-compartmental analysis of strasseriolide **D** in mouse plasma (after i.v.  bolus input). **Table S7.** In vivo mice efficacy data of strasseriolide **C** at i.p. dosage of 50 mg/kg. **Table S8.** In vivo mice efficacy data of strasseriolide **D** at i.p. dosage of 22 mg/kg.

## Data Availability

A “Supporting information” file has been uploaded as an additional file to this manuscript.

## Abbreviations

MTT: 3-(4, 5-Dimethylthiazol-2-yl)-2,5-diphenyltetrazolium bromide
hERG: Human *Ether-à-go-go*-related gene
HEK: Human embryonic kidney
HPMC: Hydroxypropyl methylcellulose
LC–MS: Liquid chromatography–mass spectrometry
LC–MS/MS: Liquid chromatography–mass spectrometry–mass spectrometry
IVIS: In vivo Imaging Software
PK: Pharmacokinetic
GFP: Green fluo rescence protein
BD: Becton, Dickinson and Company
MEM: Minimum essential medium
DMSO: Dimethyl sulfoxide
PBS: Phosphate-buffered saline.
HEPES: 4-(2-Hydroxyethyl)-1-piperazineethanesulfonic acid
IC_50_: Half maximal inhibitory concentration
NADPH: Nicotinamide dinucleotide phosphate (reduced)
HLM: Human liver microsomes
G6P: Glucose-6-phophate
G6PDH: Glucose-6-phophate dehydrogenase
Cmax: Maximum concentration
SD: Standard deviation
