# Supplementary material for: Preclinical evaluation of strasseriolides A–D, potent antiplasmodial macrolides isolated from Strasseria geniculata CF-247,251
Source: Malar J. 2021 Dec 5;20:457. doi: 10.1186/s12936-021-03993-8 (PMC8647499; doi:10.1186/s12936-021-03993-8)
Supplement: Supplementary file 1 — Additional file 1: Fig. S1. Calibration curve of test strasseriolide B (i.e. peak area ration of strasseriolide B:C vs concentration of B). Table S1. Calibration curve table of strasseriolide B. Table S2. QC table used to calculate the precision and accuracy of the method. Table S3a. Strasseriolide A quantification in mouse plasma (i.v. dosage of 25 mg/kg). Table S3b. Non-compartmental analysis of strasseriolide A in mouse plasma (after i.v. bolus input). Table S4. Strasseriolide B quantification in mouse plasma (i.v. dosage of 25 mg/kg). Table S5a. Strasseriolide C quantification in mouse plasma (i.v. dosage of 25 mg/Kg). Table S5b. Non-compartmental analysis of strasseriolide C in mouse plasma (after i.v. bolus input). Table S6a. Strasseriolide D quantification in mouse plasma (i.v. dosage of 25 mg/Kg). Table S6b. Non-compartmental analysis of strasseriolide D in mouse plasma (after i.v. bolus input). Table S7. In vivo mice efficacy data of strasseriolide C at i.p. dosage of 50 mg/kg. Table S8. In vivo mice efficacy data of strasseriolide D at i.p. dosage of 22 mg/kg. [file 12936_2021_3993_MOESM1_ESM.pdf]

Additional information

Preclinical evaluation of strasseriolides A-D, potent antiplasmodial macrolides isolated from *Strasseria geniculata* CF-247251

Frederick Annang[1,†], Guiomar Pérez-Moreno[2,†], Caridad Díaz[1], Victor González-Menéndez[1], Nuria de Pedro Montejo[1], José Pérez del Palacio[1], Paula Sánchez[2], Scott Tanghe[3], Ana Rodriguez[3], Ignacio Pérez-Victoria[1], Juan Cantizani[1], Luis M. Ruiz-Pérez[2], Olga Genilloud[1], Fernando Reyes[1], Francisca Vicente[1]*, Dolores González-Pacanowska[1]*

[1]Fundación MEDINA, Avda. del Conocimiento 34, 18016, Armilla, Granada, Spain.

[2]Instituto de Parasitología y Biomedicina "López-Neyra", Consejo Superior de Investigaciones Científicas (CSIC) Avda. del Conocimiento 17, 18016, Armilla, Granada, Spain.

[3]Department of Microbiology, New York University School of Medicine, New York, NY 10016, USA

*francisca.vicente@medinaandalucia.es

*dgonzalez@ipb.csic.es

[†]Equal contribution

LC-MS method development for the quantification of compounds in mouse plasma

The following LC-MS method was developed for the quantification and validation of strasseriolide **B** in mice plasma and was adapted for the quantification of strasseriolides **A**, **C**, **D**.

The mass spectrometer used in this experiment was operated in positive ion electrospray ionization (ESI) mode for strasseriolides **A** and **D** and in negative ion ESI mode for strasseriolide **B** and **C**. The LC-MS instrumentation used was composed of an API 4000 mass spectrometer (Applied Biosystems/AB SCIEX, ThermoFisher Scientific, USA) coupled to an Agilent 1290 HPLC system (Agilent Technologies, USA) equipped with an analytical supelco C18 column of 2.1 x 50 mm, 5 µm and auto sampler CTC Pal-xt (CTC Analytics AG, Switzerland). Mobile phase A [90 % water/Acetonitrile (v/v) + 0.1 % formic acid], B [90 % Acetonitrile/water (v/v) + 0.1 % formic acid], rinse solution A [Acetonitrile: methanol: water (20: 40: 40) % v/v + 0.1 % formic acid], B (Water + 0.1% formic acid), and reconstitution solution [Water: acetonitrile (50:50) % v/v] were also used. Five microlitres (5 µL) sample injection and 0.6 mL/min flow rate were used with the following mobile phases B/A elution conditions: gradient maintained at 5% B from 0.0-0.5 min, then increased linearly to 95% B from 0.5-2.10 min, maintained at 95% B from 2.10-3.20 min, lowered to 5% B from 3.20-3.30 min and later maintained at 95% B from 3.30-4.50 min to wash the column. The auto-sampler was set at 4 ^{o}C to rinse the injection system in between samples by using the rinsing solutions A and B respectively.

Ten millimolar (10 mM) DMSO stocks of the test compound, strasseriolide **B** (negative *m/z* 435 monitored) and internal standard, strasseriolide **C** (negative *m/z* 449 monitored) were initially prepared, from which 9.94 µM (4333 ng/mL) working concentrations were made in DMSO. An aliquot of the strasseriolide **B** working concentration was diluted (1:3) in DMSO to prepare a duplicate six-point calibration curve from 0.0016 µM (0.7 ng/mL) to 1.17 µM (510 ng/mL). Four quality control concentrations of strasseriolide **B** at 1.18 (QC1), 0.294 (QC2), 0.0092 (QC3) and 0.00459 µM (QC4) (i.e. 513, 128.0, 4.0 and 2.0 ng/mL) were also prepared in duplicate.

Blank mouse plasma (prepared from non-injected mouse) was retrieved from the freezer (-80 °C) and kept on ice to thaw. The sample was vortexed and 15 µL aliquots transferred into 0.7 mL Eppendorf tubes after which 35 µL Milli-Q water were added and vortexed. Duplicate sets of the diluted plasma were then respectively spiked with 2 uL of each concentration of the calibration curve. Another duplicate set of the diluted plasma was spiked with 2 uL of each QC concentration. All the samples were also spiked with 2 µL internal standard (i.e. strasseriolide **C** at final concentration of 0.222 µM). Four microlitres (4 µL) DMSO were added to a set of six diluted plasma to serve as blanks. Two microlitres (2 µL) QC/internal standard (both at final conc of 0.222 µM) were added to 100 µL aliquots of reconstitution solution to serve as non-matrix samples.

One hundred and sixty-two microlitres (162 µL) ice-cold acetonitrile was added to all the plasma-containing samples, centrifuged at 13300 rpm for 15 min at 4 °C. Aliquots of 180 µL were carefully transferred (without disturbing the protein precipitate) from each

tube into clean HPLC vials and evaporated to dryness in an EZ2 Genevac set at room temperature. The dried samples were re-dissolved in 40 µL reconstitution solution by vortexing. The samples were placed in an auto-sampler mass spectrometer, subjected to LC-MS-MS and the resulting data analysed by Analyst® software version 1.5.2. A standard calibration curve was drawn by plotting the peak area ratio of test compound to internal standard against the concentrations of the calibration curve.

The following qualitative data were validated in this method development:
(1) Accurate metabolite identification – that the plasma ionic peak of strasseriolide **B** (*m/z* 435 and retention time of 2.42 min) and that of the internal standard, strasseriolide **C** (*m/z* 449 and retention time of 2.26 minutes) corresponded well with what is known for the pure compounds.
(2) Specificity - this was evaluated by the inclusion of six blank plasma samples (only spiked with DMSO), from which no significant interferences were observed at the retention times of the test compound and internal standard.
(3) Auto-sampler carryover was absent - implying that when samples are auto-injected into the LC-MS system in a pre-programmed sequence, there is no interference nor mixing between samples.
(4) Linearity of calibration curves - two independent standard calibration curves (0.0016 to 1.17 µM) of test compound (strasseriolide **B**), were prepared in mouse plasma and analyzed. The linearity of this curve was greater than 0.995 over the range of concentrations analyzed (**Fig. S1** and **Table S1**). The peak area ratio of test compound (strasseriolide **B)** to the internal standard (strasseriolide **C**), was correlated to the test

compound concentration using the linear fit with $1/X^2$ weighting (where X is concentration). All the standard calibration curve concentrations were recomputed from the curve plotted and were found to be of acceptable precision (CV ± 20 %) and accuracy (± 20 %) when compared to the respective theoretical concentrations (**Table S1**) except for the lowest concentration of 0.00 16 µM which was no included in plotting the curve. Hence, the lower and upper concentration limits of the calibration curve were established at 0.00484 and 1.18 µM respectively. The overall precision and accuracy of the developed method were determined using the three QC concentrations shown on **Table S2**.

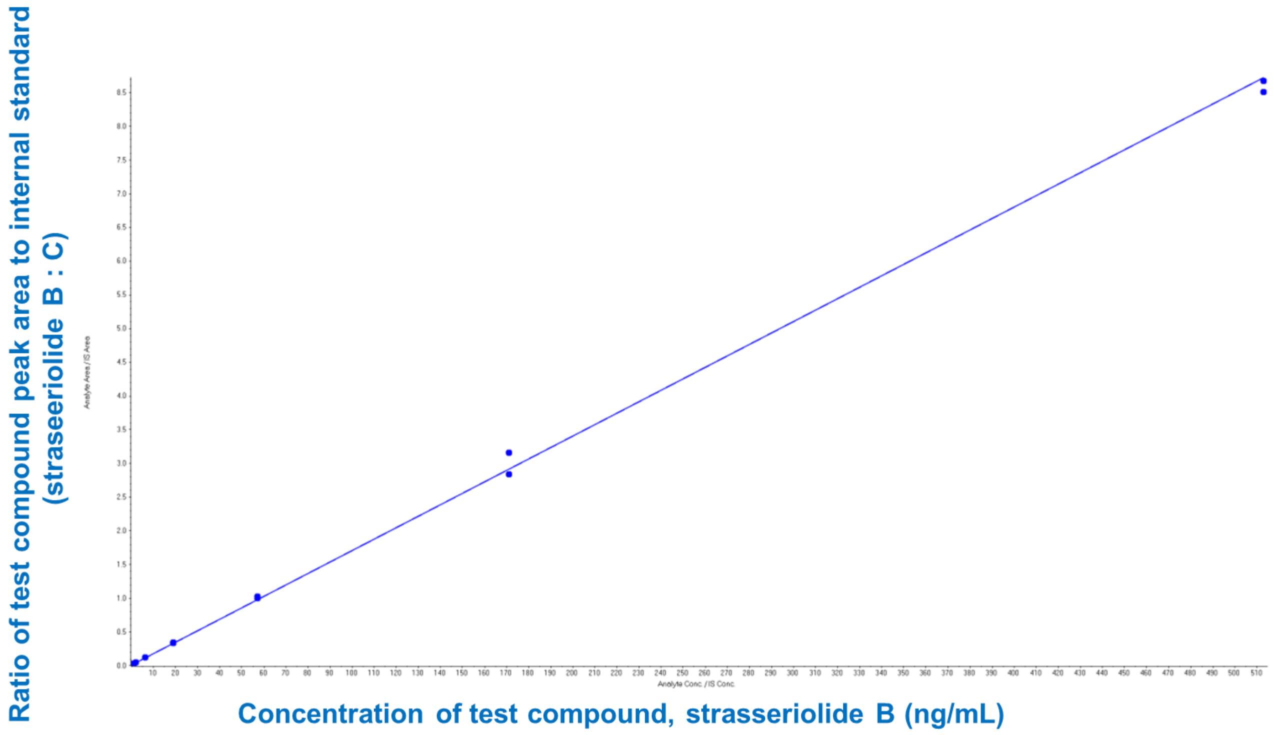

Fig. S1. Calibration curve of test strasseriolide **B** (i.e. peak area ration of strasseriolide **B**:**C** vs concentration of **B)**

The regression equation for the curve is:

Y= 2.16 x 10^{-2} + 0.0178X + 0.00594 (r = 0.9997), where X is the expected concentration (**Table S1**) and Y is the peak area ratio of test compound (strasseriolide **B**) to the internal standard (strasseriolide **C**).

Table S1. Calibration curve table of strasseriolide **B**.

**Expected conc. in ng/mL (or µM)**	**No. of replicates**	**Experimental mean conc. in ng/mL (or µM)**	**% Accuracy**	**Std. deviation**	**% CV**
2.11 (0.00484)	2	2.40 (0.00550)	113.8	0.04	1.6
6.33 (0.0145)	2	6.48 (0.0147)	102.3	0.05	0.7
19 (0.0436)	2	18.69 (0.0429)	98.4	0.25	1.3
57 (0.131)	2	56.85 (0.130)	99.7	0.84	1.5
171 (0.392)	2	171.12 (0.392)	100.1	13.28	7.8
513 (1.177)	2	512.99 (1.177)	100.0	7.28	1.4
0.7 (0.0061)	1	0.55 (0.00126)	78.0	NA	NA

Table S2. QC table used to calculate the precision and accuracy of the method.

Compound	Expected conc. in ng/mL (or µM))	No. values	Experimental mean conc. in ng/mL (or µM)	STD deviation	% CV	% Accuracy
**QC3**	4 (0.00917)	5	3.606 (0.00827)	0.211	5.84	90.162
**QC2**	128 (0.294)	5	117.3 (0.269)	5.273	4.49	91.686
**QC1**	513 (1.177)	5	506.9 (1.163)	12.985	2.56	98.817

(5) The mean recovery rate for the test compound (strasseriolide **B)** – this was calculated to be 29 % by comparing the plasma QC peak area ratios at the established retention time to peak area ratios of non-matrix samples at the same retention time.

Having validated the LC-MS method, it was used with some adjustments to quantify the mice plasma concentrations of strasseriolides **A-D** after intravenous dosage at 25 mg/kg as described in the methods section of the main text.

Computing *in vivo* (mice) PK parameters of strasseriolides A-D

After intravenous injection of mice with each of the test compounds and LC-MS quantification of the compounds in the plasma at different time points (described in the

main text), the data was fed into the *PK solver 2.0* add-in program in an Excel file with "non-compartmental analysis of plasma data after intravenous bolus input" and from the concentration vs time graph, the PK parameters of each compound were computed.

Table S3a. Strasseriolide **A** quantification in mouse plasma (i.v. dosage of 25 mg/kg)

Sample Name	Calc. Conc. (ng/ml)	Calc. Conc. (µM)*	Ave. Conc. (µM)	STD deviation	Analyte RT (min)
T 24 h (1440 minutes) 1	<2.1	<0.00484	<0.00484	0	2.52
T 24 h (1440 minutes) 2	<2.1	<0.00484			2.43
T 24 h (1440 minutes) 3	<2.1	<0.00484			2.82
T 6 h (360 minutes) 1	3.19	0.00735	0.01156	0.0086	2.61
T 6 h (360 minutes) 2	2.53	0.00583			2.61
T 6 h (360 minutes) 3	9.33	0.02150			2.61
T 2 h (120 minutes) 1	43.7	0.1007	0.0935	0.0374	2.62
T 2 h (120 minutes) 2	55.0	0.1267			2.61
T 2 h (120 minutes) 3	23.0	0.0530			2.61
T 0.5 h (30 minutes) 1	343.0	0.790	0.498	0.290	2.62
T 0.5 h (30 minutes) 2	214.0	0.493			2.61
T 0.5 h (30 minutes) 3	91.6	0.2111			2.61

*Molecular weight of strasseriolide **A** = 434 g/mol

Table S3b. Non-compartmental analysis of strasseriolide **A** in mouse plasma (after i.v. bolus input)

Parameter	Unit	Value
Lambda_z	1/h	0.157078075
t1/2*	h	4.412755773
Tmax*	h	0.5
Cmax*	ng/ml (or μM)	216.2 (or 0.498)
C0*	ng/ml (or μM)	377.5422633 (or 0.870)
Clast_obs/Cmax		0.009713228
AUC 0-t	ng/ml x h (or μM x h)	496.1355658 (or 1.143)
AUC 0-inf_obs	ng/ml x h (or μM x h)	509.5047136 (or 1.174)
AUC 0-t/0-inf_obs		0.973760502
AUMC 0-inf_obs	ng/ml x h^2 (or μM x h^2)	1520.971031 (or 3.505)
MRT 0-inf_obs	h	2.98519521
Vz_obs	(mg/kg)/(ng/ml)	0.312374974 (or 312.4 L/kg)
Cl_obs	(mg/kg)/(ng/ml)/h	0.049067259
Vss_obs	(mg/kg)/(ng/ml)	0.146475348

* The parameters Cmax, C0 and Tmax obtained may not be the accurate values since blood samples were not collected at time T0 (or at times close enough to T0) in our experiment.

- λz (Lambda-z) – Individual estimate of the terminal elimination rate constant, calculated using log-linear regression of the terminal portions of the plasma concentration-versus-time curves.
- t1/2 – Apparent terminal elimination half-life time, defined as 0.693 / λz
- C-max – Maximum concentration achieved
- T-max – Time to reach maximum concentration
- AUC0-t – The area under the concentration vs. time curve, calculated as sum of AUCs using linear trapezoidal summation from time 0 to the last measurable data point
- AUC0-inf – The area under the plasma concentration-time curve extrapolated to infinity, calculated as: AUC0-inf =AUC0-t + Clast / λz, where Clast is the last measurable concentration.
- MRT – Mean retention time
- Vss – Steady state volume
- Vz_obs – Plasma distribution volume
- Cl – Clearance

Table S4. Strasseriolide **B** quantification in mouse plasma (i.v. dosage of 25 mg/kg)

**Sample Name**	**Calc. Conc. (ng/ml)**	**Calc. Conc. (µM)***	**Ave. Conc. (µM)**	**STD deviation**	**Analyte RT (min)**
T 8 minutes 1**	8110.0	18.6	19.6	1.2	2.39
T 8 minutes 2**	9098.0	20.9			2.39

Sample Name	Calc. Conc. (ng/ml)	Calc. Conc. (µM)*	Ave. Conc. (µM)	STD deviation	Analyte RT (min)
T 8 minutes 3**	8419.0	19.3			2.39

*Molecular weight of strasseriolide **B** = 436 g/mol

**All the three mice died about eight minutes after injection with the compound

S5a. Strasseriolide **C** quantification in mouse plasma (i.v. dosage of 25 mg/Kg)

Sample Name	Calc. Conc. (ng/ml)	Calc. Conc. (µM)*	Ave. Conc. (µM)	STD deviation	Analyte RT (min)
T 24 h (1440 minutes) 1	2.04	0.00453			2.12
T 24 h (1440 minutes) 2	1.27	0.00282	0.00354	0.00089	2.12
T 24 h (1440 minutes) 3	1.47	0.00327			2.12
T 6 h (360 minutes) 1	10.4	0.0231			2.12
T 6 h (360 minutes) 2	10.6	0.0236	0.024	0.0012	2.12
T 6 h (360 minutes) 3	11.4	0.0253			2.12
T 2 h (120 minutes) 1	20.7	0.046			2.12
T 2 h (120 minutes) 2	19.8	0.044	0.040	0.009	2.12
T 2 h (120 minutes) 3	13.6	0.030			2.12
T 30 minutes 1	122.0	0.271			2.12
T 30 minutes 2	102.0	0.227	0.246	0.023	2.12

Additional information

Sample Name	Calc. Conc. (ng/ml)	Calc. Conc. (µM)*	Ave. Conc. (µM)	STD deviation	Analyte RT (min)
T 30 minutes 3	108.0	0.240			2.12

*Molecular weight of strasseriolide **C** = 450 g/mol

Table S5b. Non-compartmental analysis of strasseriolide **C** in mouse plasma (after i.v. bolus input)

Parameter	Unit	Value
Lambda_z	1/h	0.109293684
t1/2*	h	6.342060722
Tmax*	h	0.5
Cmax*	ng/ml (or µM)	110.67 (or 0.246)
C0*	ng/ml (or µM)	203.1753953 (or 0.452)
Clast_obs/Cmax		0.014324324
AUC 0-t	ng/ml x h (or µM x h)	344.6788488 (or 0.766)
AUC 0-inf_obs	ng/ml x h (or µM x h)	359.2268075 (or 0.798)
AUC 0-t/0-inf_obs		0.959502024
AUMC 0-inf_obs	ng/ml x h^2 (or µM x h^2)	1693.549878 (or 3.763)
MRT 0-inf_obs	h	4.714430667
Vz_obs	(mg/kg)/(ng/ml)	0.636760632 (or 636.8 L/kg)
Cl_obs	(mg/kg)/(ng/ml)/h	0.069593915

Additional information

Vss_obs	(mg/kg)/(ng/ml)	0.328095688

* The parameters Cmax, C0 and Tmax obtained may not be the accurate values since blood samples were not collected at time T0 (or at times close enough to T0) in our experiment.

Table S6a. Strasseriolide **D** quantification in mouse plasma (i.v. dosage of 25 mg/Kg)

Sample Name	Calc. Conc. (ng/ml)	Calc. Conc. (µM)*	Ave. Conc. (µM)	STD deviation	Analyte RT
T 24 h (1440 minutes) 1	49.2	0.109	0.107	0.005	2.08
T 24 h (1440 minutes) 2	49.9	0.110			2.08
T 24 h (1440 minutes) 3	46.1	0.102			2.08
T 5 h (300 minutes) 1	195.0	0.431	0.389	0.037	2.08
T 5 h (300 minutes) 2	164.0	0.363			2.08
T 5 h (300 minutes) 3	168.0	0.372			2.08
T 1.5 h (90 minutes) 1	307.0	0.679	1.247	0.497	2.08
T 1.5 h (90 minutes) 2	658.0	1.456			2.08
T 1.5 h (90 minutes) 3	726.0	1.606			2.08

*Molecular weight of strasseriolide **D** = 452 g/mol

Table S6b. Non-compartmental analysis of strasseriolide **D** in mouse plasma (after i.v. bolus input)

Parameter	Unit	Value
Lambda_z	1/h	0.09534357
t1/2*	h	7.269993954
Tmax*	h	1.5
Cmax*	ng/ml (or µM)	564 (or 1.247)
C0*	ng/ml (or µM)	929.0437116 (or 2.055)
Clast_obs/Cmax		0.085815603
AUC 0-t	ng/ml x h (or µM x h)	4546.582784 (or 10.06)
AUC 0-inf_obs	ng/ml x h (µM x h)	5054.22058 (or 11.18)
AUC 0-t/0-inf_obs		0.899561606
AUMC 0-inf_obs	ng/ml x h^2 (µM x h^2)	40557.80737 (or 89.73)
MRT 0-inf_obs	h	8.024542405
Vz_obs	(mg/kg)/(ng/ml)	0.051879336 (or 51.9 L/kg)
Cl_obs	(mg/kg)/(ng/ml)/h	0.004946361
Vss_obs	(mg/kg)/(ng/ml)	0.039692284

* The parameters Cmax, C0 and Tmax obtained may not be the accurate values since blood samples were not collected at time T0 (or at times close enough to T0) in our experiment.

Calculation of theoretical Cmax achievable with 25 mg/kg drug dosage in mice

For purpose of comparison with the experimental Cmax values, the theoretical Cmax achievable with the drug dosage of 25 mg/kg (0.5 mg drug in 200 µL vehicle vehicle) was calculated with two key ideal-situation assumptions: (i) total mouse plasma volume is estimated in the literature to be 1.8 mL (1), (ii) that theoretically all the compounds are totally soluble in the vehicle as well as the whole mouse plasma volume. From the above assumptions, the theoretical maximum concentration (theoretical Cmax) achievable in the mouse plasma could be calculated as 0.5 mg/2 mL (i.e 1.8 mL plasma + 0.2 mL vehicle). This concentration is equivalent to 250000 ng/mL or 250000 ppb (i.e. between 553-573 µM for all the four compounds).

Table S7. *In vivo* mice efficacy data of strasseriolide **C** at i.p. dosage of 50 mg/kg

	**Parasitaemia** (Luminescence arbitrary units)				
	Replicate 1	Replicate 2	Replicate 3	Replicate 4	Average ± SD
Vehicle only	2.90×10^6	1.20×10^7	2.39×10^6	5.57×10^6	$5.72 \times 10^6 \pm 4.43 \times 10^6$
Strasseriolide **C**	6.98×10^6	2.22×10^6	2.90×10^6	2.55×10^6	$3.66 \times 10^6 \pm 2.23 \times 10^6$
Chloroquine	0.00	0.00	0.00	0.00	0.00

Table S8. *In vivo* mice efficacy ta daof strasseriolide **D** at i.p. dosage of 22 mg/kg

	**Parasitaemia** (Luminescence arbitrary units)					
	Replicate 1	Replicate 2	Replicate 3	Replicate 4	Replicate 5	Average ± SD
Vehicle only	7.81×10^5	7.45×10^5	6.06×10^5	4.02×10^4	3.64×10^5	$5.07 \times 10^5 \pm 3.08 \times 10^5$
Strasseriolide **D**	2.46×10^5	1.59×10^5	1.88×10^5	2.56×10^4	6.93×10^3	$1.25 \times 10^5 \pm 1.04 \times 10^5$
Chloroquine	3.86×10^3	2.17×10^3	2.63×10^3	3.38×10^3	-	$3.01 \times 10^3 \pm 0.755 \times 10^3$

References

1. Riches, A. C., Sharp, J. G., Thomas, D. B., Smith, S. V.: Blood volume determination in the mouse. J Phygiol **228**, 279-284 (1973)
